# Polyvascular Disease in Patients Presenting with Acute Coronary Syndrome: Its Predictors and Outcomes

**DOI:** 10.1100/2012/284851

**Published:** 2012-01-04

**Authors:** Hassan Al Thani, Ayman El-Menyar, Khalid F. AlHabib, Ahmed Al-Motarreb, Ahmad Hersi, Hussam AlFaleh, Nidal Asaad, Shukri Al Saif, Wael Almahmeed, Kadhim Sulaiman, Haitham Amin, Alawi A. Alsheikh-Ali, Khalid AlNemer, Jassim Al Suwaidi

**Affiliations:** ^1^Department of Cardiology and Cardiovascular Surgery, Hamad General Hospital, Doha 3050, Qatar; ^2^Department of Clinical Medicine, Weill Cornell Medical College, Doha 24144, Qatar; ^3^Department of Cardiology, King Fahad Cardiac Centre, College of Medicine, King Saud University, Riyadh 11472, Saudi Arabia; ^4^Faculty of Medicine, Sana'a University, Sana'a, Yemen; ^5^Department of Cardiology, Saud AllBabtain Cardiac Centre, Dammam 11850, Saudi Arabia; ^6^Institute of Cardiac Sciences, Sheikh Khalifa Medical City, Abu Dhabi, UAE; ^7^Department of Cardiology, Royal Hospital, Muscat, Oman; ^8^Mohammed Bin Khalifa Cardiac Centre, Bahrain; ^9^Tufts Clinical and Translational Science Institute, Tufts University School of Medicine, Boston, MA 02111, USA; ^10^Department of Cardiology, Security Forces Hospital, Riyadh, Saudi Arabia

## Abstract

We evaluated prevalence and clinical outcome of polyvascular disease (PolyVD) in patients presenting with acute coronary syndrome (ACS). Data for 7689 consecutive ACS patients were collected from the 2nd Gulf Registry of Acute Coronary Events between October 2008 and June 2009. Patients were divided into 2 groups (ACS with versus without PolyVD). All-cause mortality was assessed at 1 and 12 months. Patients with PolyVD were older and more likely to have cardiovascular risk factors. On presentation, those patients were more likely to have atypical angina, high resting heart rate, high Killip class, and GRACE risk scoring. They were less likely to receive evidence-based therapies. Diabetes mellitus, renal failure, and hypertension were independent predictors for presence of PolyVD. PolyVD was associated with worse in-hospital outcomes (except for major bleedings) and all-cause mortality even after adjusting for baseline covariates. Great efforts should be directed toward primary and secondary preventive measures.

## 1. Introduction

Atherothrombosis is a systemic disease that often occurs at more than one vascular site and should be considered in practice as an integral disease [1–3]. Moreover, patients with affected arterial disease are more likely to develop higher event rates than patients with multiple risk factors only [3, 4]. Polyvascular disease (PolyVD) defined as presence of more than one affected vascular bed, that is, any combination of the following: coronary artery disease (CAD), peripheral arterial disease (PAD), and cerebrovascular disease (CVD) [5–9]. The frequency and impact of PolyVD in patients with acute coronary syndrome (ACS) in the Gulf region of the Middle East have not been studied yet. These countries have higher prevalence of the traditional risk factors in a unique fashion [1]. The aim of the current study is to evaluate the prevalence of PolyVD and its impact on the in-hospital major adverse events and 1-year mortality across ACS in a Middle Eastern population.

## 2. Methods

### 2.1. Study Population

Data were collected from a prospective, multicenter study of the 2nd Gulf Registry of Acute Coronary Events (Gulf RACE-2) between October 2008 and June 2009. We recruited 7,930 consecutive patients with ACS from 6 adjacent Middle Eastern Gulf countries (Bahrain, Kingdom of Saudi Arabia, Qatar, Oman, United Arab Emirates, and Yemen). Patients diagnosed with ACS, including unstable angina (UA) and non-ST- and ST-elevation myocardial infarction (NSTEMI and STEMI, resp.), were enrolled from 65 hospitals. On-site cardiac catheterization laboratory was available in 43% of the participating hospitals. All prospective patients with ACS were eligible for enrollment. The study received ethics approval from the institutional review boards in all participating hospitals. Full details of the methods have been previously published [10].

### 2.2. Definitions

*ACS:* diagnosis of the different types of ACS and definitions of data variables were based on the American College of Cardiology clinical data standards [10, 11]. Peripheral arterial disease (PAD): defined as presence of *any of the* following: intermittent claudication, critical limb ischemia (ulcer or gangrene), peripheral bypass surgery (surgical bypass for PAD indication), or peripheral percutaneous transluminal angioplasty. *Polyvascular disease* (PolyVD) was defined as presence of more than one affected vascular bed, that is, CAD, cerebrovascular disease (CVD), and asymptomatic or symptomatic peripheral arteries (PAD) [6]. A case report form (CRF) for each patient with suspected ACS was filled out upon hospital admission by assigned physicians and/or research assistants using standard definitions and was completed throughout the patient's hospital stay. All CRFs were verified by a cardiologist then sent online to the principal coordinating center, where the forms were further checked for mistakes before submission for final analysis.

### 2.3. Statistical Analysis

Data are presented as proportions or mean ± standard deviation (SD) as appropriate. Baseline demographic characteristics, past medical history, clinical presentation, and clinical outcomes were compared between 2 groups (ACS with versus without PolyVD). Subanalysis was performed comparing the clinical outcomes among various combinations of vascular bed affection (ACS alone, ACS plus PAD, ACS plus CVD, and ACS plus PAD and CVD). Statistical analyses were conducted using the Student's *t*-test for continuous variables and Pearson chi-square (*χ* ^2^) test for categorical variables. Primary endpoints included recurrent ischemia, heart failure (HF), stroke, major bleedings, and mortality. All-cause mortality was assessed at 1-month and 12-month follow-up period. In order to assess the independent association of PolyVD with clinical outcomes, logistic regression analysis models were used and crude and adjusted odd ratios were calculated. We included significant baseline variables in the analysis such as age, sex, diabetes mellitus, hypertension, dyslipidemia, smoking, renal failure, prior coronary artery disease and prior coronary revascularization in addition to the type of ACS at presentation. Univariate and multivariate analysis for clinical outcomes were tested. Global Registry of ACS events (GRACE) risk scores for hospital mortality were used to stratify the risk status of patients at presentation as low, intermediate, or high [12]. All *P* values were the results of 2-tailed tests and values <0.05 were considered significant. Data analysis was carried out using the Statistical Package for Social Sciences version 18 (SPSS Inc., USA).

## 3. Results

### 3.1. Clinical and Biochemical Profiles

Out of the 7689 patients who were admitted with ACS, PolyVD was documented in 428 patients (5.6%) in terms of ACS plus PAD (110 patients), ACS plus CVD (284 patients), and ACS plus PAD and CVD (34 patients).  Table 1 shows the baseline characteristics and risk factors of patients with PolyVD including ACS in comparison to ACS-alone patients. Patients with PolyVD were 6 years older (62 ± 11 versus 56 ± 12, *P* < 0.0001) and were more likely to be female (31% versus 20%, *P* = 0.001). PolyVD patients were more likely to have risk factors such as diabetes mellitus (67% versus 38%, *P* = 0.001), hypertension (78% versus 45%, *P* = 0.001), dyslipidemia (55% versus 36%, *P* = 0.001), and renal failure (14% versus 3%, *P* = 0.001). They were less likely to be smokers (45% versus 54%, *P* = 0.001). On presentation with ACS, PolyVD patients had higher heart rate, and GRACE risk score and were more likely to present with a higher Killip class (*P* = 0.001 for all). NSTEACS was the most frequent diagnosis in PolyVD patients whereas STEMI was the predominant diagnosis in ACS-alone group.

### 3.2. In-Hospital Treatment Pattern

Table 2 demonstrates the treatment pattern for patients with and without PolyVD. In regard to on-admission therapy, there were no differences between the two groups in the use of heparin and glycoprotein inhibitors. Apart from thrombolysis therapy use that was in favor of ACS-alone group, patients with PolyVD were more likely to be treated with oral antiplatelet, angiotensin-converting enzyme inhibitors, statin, and *β*-blockers. Coronary intervention was more frequently used in ACS-alone group in comparison to PolyVD patients. Aspirin, statin, and *β*-blockers were more frequently used during hospitalization and at discharge in ACS-alone patients.

### 3.3. Predictors for Polyvascular Disease

Multivariate logistic regression analysis showed that the important independent predictors for the presence of PolyVD in ACS patients were diabetes mellitus (adjusted OR 2.28; 95% CI 1.81–2.89), renal failure (adjusted OR 2.32; 95% CI 1.54–3.03) and hypertension (adjusted OR 2.66; 2.03–3.49), *P* = 0.001 for all (Table 3).

### 3.4. Clinical Outcomes

**Figure 1:**
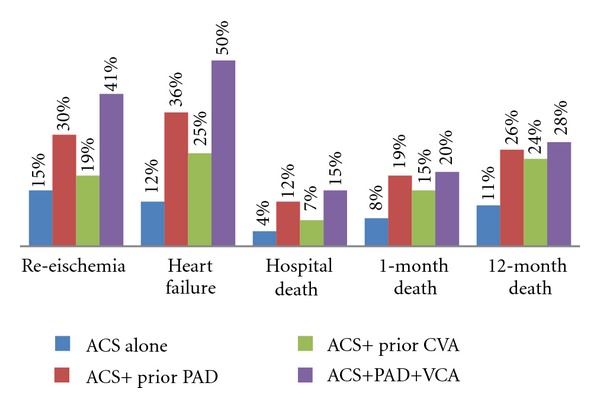
Clinical outcomes in different vascular disease combinations (*P* = 0.001 for all comparisons).

**Figure 2:**
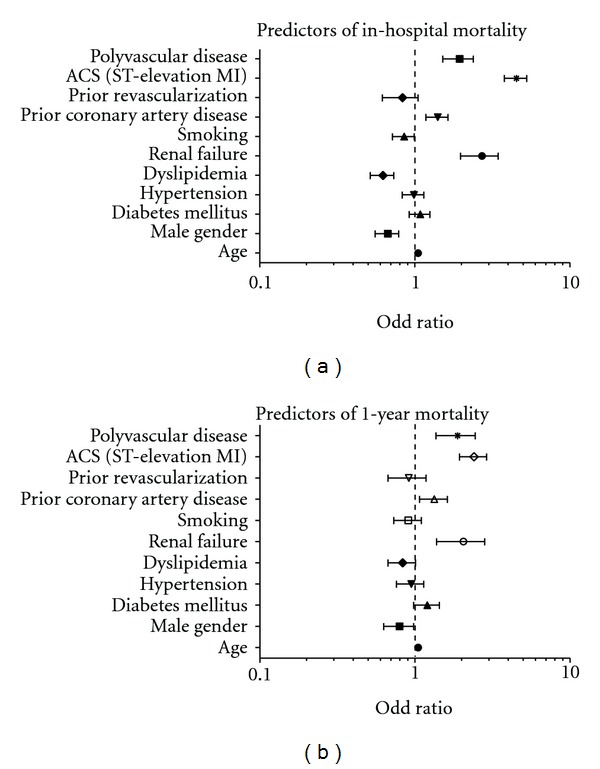
Clinical predictors for in-hospital and 1-year mortality.

Figure 1 demonstrates the clinical outcomes in different vascular disease combinations. Table 2 shows hospital outcomes in PolyVD versus ACS-alone group. All primary endpoints were significantly worse in PolyVD except for major bleedings. One- and 12-month all-cause mortality was 2-times greater in PolyVD group. Univariate analysis showed that PolyVD was predictor for reischemia, HF, stroke, and hospital, 1, and 12-month mortality (*P* = 0.001 for all). Similarly, multivariate logistic regression analysis denoted that PolyVD was an independent predictor for all outcomes except for major bleeding. PolyVD was strong predictor for in-hospital stroke (adjusted OR 5.40; 95% CI 2.17–13.29, *P* = 0.001  Table 4 and Figure 2).

### 3.5. Gender and Polyvascular Disease

In comparison to their counterparts with ACS alone, men and women with PolyVD were older and had higher percent of commodities except for smoking. When compared to men, women with ACS alone had significant worse outcomes in terms of re-ischemia (18% versus 14%), in-hospital mortality (6.3% versus 3.7%), 1-month mortality (9.7% versus 6.8%), *P* = 0.001 for all. In all forms of PolyVD, there were no significant morbidity and mortality differences between men and women (Table 5).

Table 6 shows risk factors and in-hospital mortality in patients with versus without polyvascular disease presenting with acute coronary syndrome in 3 major clinical studies.

## 4. Discussion

The present study reports on the frequency, predictors and implication of PolyVD in patients presenting with ACS in the Gulf Region of the Middle East. Up to our knowledge, PolyVD in ACS patients was not reported in the Middle East before. There are several key findings in this study. *First*, PolyVD is a common disorder in that region in the setting of acute coronary events and represents a marker of high-risk population. The prevalence of PolyVD in the current study (5.6%) is over 2-fold lower versus GRACE study (15.6%), Alliance project (13%), and MASCARA study (16.6% Table 6). Among the 4 studies, the high percent of young age, DM, and renal failure were observed in the current study. However, the difference in presentations and outcomes in the 4 studies may in part relate to diversity of biological and environmental factors between the different ethnicities. This observation needs further confirmatory studies. *Second*, PolyVD is associated with higher mortality rate even after adjusting for baseline variables. *Third*, at presentation, those patients were more likely to have atypical presentation, high resting heart rate, high Killip class, and high GRACE risk score. Moreover, NSTEACS was the most frequent diagnosis in PolyVD in comparison to ACS-alone group. Our findings are consistent with data from Western reports [5–9]. These studies showed that the management of PolyVD group was less aggressive in terms of less in-hospital coronary intervention and less evidence-based therapies during hospitalization and at discharge [6–8]. Several factors could explain the undertreatment trend in PolyVD group such as high percent of comorbidities with subsequent contraindication of some medications, physician and patient discretion, and possible socioeconomic factors. Understanding such reasons may reduce this therapy imbalance. *Fourth*, the current study shows that increasing the number of the affected vascular bed is associated with the worst clinical scenario (Figure 1). Apart from major bleedings, PolyVD is independent predictor for all in-hospital adverse outcomes. PolyVD increased risk of stroke 5-times even after adjustment for other covariates. PAD increased rate of HF and hospital mortality by 3-times whereas presence of CVD increased rate of in-hospital stroke 4-times. Data from Table 5 indicates that the highest mortality rate was observed in patients with PAD in our study, in patients with CVD in ALLIANCE project, and in patients with triple vascular bed affection in MASCARA and our study. Data from the Japanese REACH registry [13] denoted that the rates of 1-year stroke and myocardial infarction were higher for patients with CVD and PAD than for patients with CVD and CAD; however, that study was not carried out during the acute setting of coronary artery disease and did not report on the in-hospital rate of stroke. *Fifth*, the present study expands the previous reports and shows that diabetes mellitus, hypertension, and renal failures are independent predictors for the presence of PolyVD. This finding highlights the importance of primary and secondary prevention in this high-risk population. *Sixth*, male gender has better outcomes in ACS alone in comparison to female. These unfavorable outcomes associated with female gender were shown in our previous work as well [14]. Interestingly, presence of PolyVD is not associated with significant differences in the outcomes between men and women, this finding warrants further exploration.

## 5. Limitation

The current study is an observational study; however, well-designed observational studies may provide valid results. Another limitation of the current retrospective analysis is that the diagnosis of PAD relied on the clinical history and not on the measurement of ankle-brachial index (ABI). This limitation could be explained in part by facts that clinical variables used are incompletely sensitive to identify PAD, approximately half of subjects with ABI <0.90 are asymptomatic, and of those that are symptomatic, only a minority have classic intermittent claudication. The presence of PolyVD is underdiagnosed in our daily practice that may underestimate its true prevalence and impact on the outcome.

## 6. Conclusion

Although PolyVD's patients are high-risk population in the setting of ACS, they received less aggressive therapy. Apart from major bleedings, PolyVD is an independent predictor for adverse hospital outcomes and short- and long-term mortality. Great efforts should be directed to primary and secondary prevention. Looking for the other affected vascular bed in ACS patients will add an important step in risk stratification and management.

## Figures and Tables

**Table 1:** Risk factors and clinical and laboratory findings in patients with acute coronary syndrome with and without polyvascular disease.

	ACS alone (*n* = 7261)	Polyvascular disease (*n* = 428)	*P* value
Age	56 ± 12	62 ± 12	0.001
Female	20	31	0.001
Diabetes mellitus	38	67	0.001
Hypertension	45.3	78	0.001
Dyslipidemia	36	55	0.001
Smoking	54	45	0.001
Renal failure	3	14	0.001
Prior coronary artery disease	39	64	0.001
Prior revascularization	11	25	0.001
Khat chewing	19	20	0.92

*Clinical presentation*			
Atypical presentation ACS	15	28	0.001
Heart rate	84 ± 20	90 ± 22	0.001
Systolic blood pressure	135 ± 29	140 ± 35	0.01
Diastolic blood pressure	81 ± 17	79 ± 20	0.02
Unstable angina	24	24	
Non-STEMI	29	45	0.001
STEMI	47	31	
Killip class > 1	21	45	0.001
Low GRACE risk score	41	19	0.001
High GRACE risk score	20	43	

*Laboratory findings*			
Fasting blood sugar (mmol/L)	7.3 ± 3	8.1 ± 3	0.001
First hemoglobin (g/dL)	13.7 ± 2	12.5 ± 2	0.001
Serum creatinine (umol/L)	100 ± 72	130 ± 110	0.001
Hemoglobin A1c (%)	7.6 ± 2.4	8 ± 2.4	0.15
Total cholesterol (mmol/L)	4.9 ± 1.6	4.4 ± 1.6	0.001
Serum triglyceride (mmol/L)	1.8 ± 1.1	1.6 ± 0.8	0.001
Low-density lipoprotein	3.2 ± 1.3	2.8 ± 1.2	0.001
High-density lipoprotein	1.05 ± 0.5	0.99 ± 0.4	0.01
Peak troponin T	1.4 ± 0.7	1.3 ± 0.6	0.02
LV ejection fraction <50%	73	85	0.001
1-vessel CAD	29	48	0.001
2-vessel CAD	25	18	0.12
3-vessel CAD	30	17	0.004

All categorical and continuous variables are given in percent and mean ± SD, respectively.

**Table 2:** Management and clinical outcomes in patients with acute coronary syndrome with and without polyvascular disease.

	ACS alone (*n* = 7261)	Polyvascular disease (*n* = 428)	*P* value
*On admission therapy %*			
Aspirin	38	74	0.001
Clopidogrel	11	23	0.001
*β*-blockers	28	43.5	0.001
ACE inhibitors	24	46	0.001
Statins	29	58	0.001
Thrombolysis*	52	20	0.001
Unfractionated heparin	42	40	0.46
LMW heparin	37	41	0.12
Glycoprotein inhibitors	7.7	8	0.82

*During hospitalization %*			
Aspirin	98.5	96	0.001
Clopidogrel	76	81	0.02
*β* blockers	75	68	0.002
ACE inhibitors	71	69	0.31
Statins	95	92.5	0.03
Coronary angiography	39	31	0.002
PCI	22	12.4	0.001

*Discharge medications %*			
Aspirin	96	92	0.001
Clopidogrel	68	70	0.32
*β* blockers	80	74	0.01
ACE inhibitors	72	63.5	0.001
Statins	92	85	0.001

*Outcomes %*			
Reischemia	15	23.4	0.001
Heart failure	11.6	29.7	0.001
Stroke	0.6	2.3	0.001
Bleedings	0.6	0.9	0.31
Hospital death	4.2	9.1	0.001
1-month death	7.4	16.3	0.001
12-month death	11.2	24.6	0.001

*ST-elevation MI, PCI: percutaneous coronary intervention.

**Table 3:** Multivariate logistic regression analysis for clinical predictors of polyvascular disease in patients presenting with acute coronary syndrome.

	Odds ratio (95% confidence interval)	*P* value
Age	1.04 (1.036–1.056)	0.001
Male gender	1.13 (0.865–1.466)	0.34
Dyslipidemia	1.10 (0.882–1.392)	0.37
Diabetes mellitus	2.28 (1.809–2.889)	0.001
Hypertension	2.66 (2.028–3.489)	0.001
Smoking	1.24 (0.970–1.591)	0.07
Obesity	1.003 (0.986–1.020)	0.62
Renal failure	2.32 (1.543–3.029)	0.001

**Table 4:** Multivariate logistic regression analysis for predictors of clinical outcomes in patients with polyvascular disease presenting with acute coronary syndrome.

	Unadjusted odds ratio (95% CI)	Adjusted odds ratio* (95% CI)
Re-ischemia	1.7 (1.37–2.15), *P* = 0.001	1.50 (1.12–2.01), *P* = 0.007
Heart failure	3.3 (2.67–4.09), *P* = 0.001	2.00 (1.47–2.61), *P* = 0.001
In-hospital Stroke	4.1 (2.03–8.22), *P* = 0.001	5.40 (2.17–13.29), *P* = 0.001
Major bleedings	1.61 (0.57–4.52), *P* = 0.36	1.68 (0.54–5.17), *P* = 0.37
Hospital death	2.3 (1.66–3.26), *P* = 0.001	1.85 (1.25–2.74), *P* = 0.01
1-month death	2.5 (1.89–3.29), *P* = 0.001	2.03 (1.46–2.83), *P* = 0.001
12-month death	2.6 (2.02–3.32), *P* = 0.001	1.83 (1.83–2.45), *P* = 0.003

CI: confidence interval, *variables adjusted for age, sex, diabetes mellitus, hypertension, dyslipidemia, smoking, renal failure, prior coronary artery disease, and prior coronary revascularization in addition to the type of ACS at presentation.

**Table 5:** Clinical profiles, admission therapy, and outcomes in men and women.

	ACS alone			Polyvascular disease		
	Men	Women	*P* value	Men	Women	*P* value
Age (yrs, mean)	55 ± 12	61 ± 12	0.001	64 ± 12	65 ± 13	0.19
Diabetes mellitus %	35	51	0.001	63	74	0.03
Hypertension %	40.5	64	0.001	76	81	0.24
Dyslipidemia %	34	44	0.001	53	63	0.55
Smoking %	65	10	0.001	57	16	0.001
Renal failure %	3	5	0.001	16	13	0.44
Admission therapy (%)						
Aspirin	98.6	98.5	0.75	96	97	0.51
Clopidogrel	79	64	0.001	82	79	0.51
Beta blockers	76	71	0.001	70	63	0.11
ACE inhibitors	71	72	0.72	70	65	0.31
Heparin	58	59	0.98	41	37	0.50
Glycoprotein inhibitors	8.3	5.2	0.001	9	6	0.23
Statin	95	94	0.01	92	94	0.57
PCI	23	19	0.01	13	10.5	0.55
Outcomes (%)						
Reischemia	14	18	0.001	21	27	0.17
Heart failure	10.7	15.5	0.001	29	34	0.28
Hospital mortality	3.7	6.3	0.001	8.6	10.8	0.46
1-month mortality	6.8	9.7	0.001	15	20	0.26
12-month mortality	10.3	15	0.001	23	29	0.22

**Table 6:** Risk factors and in-hospital mortality in patients with versus without polyvascular disease presenting with acute coronary syndrome in different clinical studies.

	GRACE (*n* = 32735) [7]				MASCARA (*n* = 6745) [9]				GULFRACE-2 (*n* = 7689)				ALLIANCE (*n* = 8904) [6]			
	A	PolyVD (15.6%)			A	PolyVD (16.6%)			A	PolyVD (5.6%)			A	PolyVD (13%)		
		B	C	D		B	C	D		B	C	D		B	C	D
Patients %	84	7	6	2	83	9	6	2	94	1	4	0.6	87	8	4	1
Age (mean yrs)	64	71	73	73	67	70	73	72	56	63	65	66	65	72		
Smoking	59	69	53	68	36/28*	61/22*	44/16*	60/21*	54	54	39	62	59	63		
Diabetes mellitus	22	38	34	42	28	49	43	52	38	77	61	82	19	34		
Hypertension	58	72	78	82	58	72	76	69	45	72	79	82	48	66		
Dyslipidemia	46	58	52	65	46	57	50	53	36	55	53	67	43	47		
Renal failure	N/A	N/A	N/A	N/A	N/A	N/A	N/A	N/A	3	20	10	30	3	12		

*Mortality*	**4.5**	**7.2**	**8.9**	**9.2**	**4.8**	**9.1**	**9.2**	**16**	**4**	**12**	**7**	**15**	**5.7**	**9.8**	**14**	**13**

A: acute coronary syndrome (ACS) alone; B: ACS plus peripheral arterial disease (PAD); C: ACS plus cerebrovascular disease (CVD); D: ACS plus PAD plus CVD; PolyVD: polyvascular disease, all categorical variables represents in percentage (%); DM: diabetes mellitus; N/A: data not available; *prior/current smoking. Data were collected from [6, 7, 9].
